# Microbial Communities Drive Methane Fluxes From Floodplain Lakes—A Hydrological Gradient Perspective

**DOI:** 10.1111/1462-2920.70127

**Published:** 2025-06-22

**Authors:** Sylwia Lew, Paweł Burandt, Katarzyna Glińska‐Lewczuk

**Affiliations:** ^1^ Department of Microbiology and Mycology University of Warmia and Mazury in Olsztyn Olsztyn Poland; ^2^ Department of Water Management and Climatology University of Warmia and Mazury in Olsztyn Olsztyn Poland

**Keywords:** floodplain lakes, greenhouse gases, methanotrophic bacteria (MOB), mGen methanogenic archaea, microbiota

## Abstract

This study examined the impact of methanotrophic bacteria and methanogenic archaea on CH_4_ fluxes from floodplain lakes at various successional stages, analysing their interactions with physicochemical properties of water. Seasonal microbiological and hydrochemical studies of 10 floodplain lakes in the Łyna River floodplain, characterised by varying hydrological connectivity, revealed that methanotrophic bacteria (MOB) and mGen significantly influenced CH_4_ and CO_2_ emissions. The microbial structure, expressed as the MOB/mGen ratio, was associated with a gradient of CH_4_ flux rates specific to each oxbow type. Average CH_4_ fluxes from the lakes were 21, 225 and 507 mg m^−2^ day^−1^ for lotic, semi‐lotic and lentic systems, respectively, while corresponding CO_2_ fluxes were 0.8, 0.7 and 1.0 g CO_2_ m^−2^ day^−1^, respectively. Statistically significant differences in CH_4_ and CO_2_ fluxes were observed between lentic and lotic water bodies. The partial least squares model indicated that water temperature significantly stimulated MOB and mGen abundances. Moreover, chlorophyll‐*a*, turbidity and chemical oxygen demand positively correlated with the presence of these microbial groups. Methanotrophs were negatively affected by NH_4_–N, while methanogens were affected by NO_3_–N. These findings highlight the complex biotic and abiotic interactions driving greenhouse gas emissions in floodplain ecosystems and suggest targeted management strategies to mitigate their climate impacts.

## Graphical abstract

This study examined the impact of methanotrophic bacteria (MOB) and methanogenic archaea (mGen) on CH_4_ emissions from oxbow lakes at various successional stages, analysing their interactions with physicochemical properties of water. The microbial structure, defined by the MOB/mGen ratio, shows emission rates of CH_4_ specific to each oxbow type. It highlighted the gradient of methane emissions due to oxbow productivity.

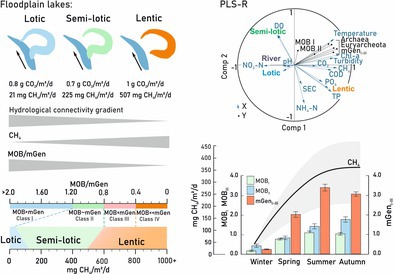

## 1 Introduction

Floodplain lakes, also known as oxbow lakes or channel cut‐offs, are a type of fluvial wetlands whose geochemical dynamics are characterised by successional processes towards overgrowth, sedimentation and organic matter accumulation (Cui et al. 2024). This makes them potential hotspots for carbon processing and exchange of large amounts of greenhouse gases (GHG), such as carbon dioxide (CO_2_) and methane (CH_4_), with the atmosphere (Colina et al. 2022). The connectivity with the parent river channel is a crucial factor regulating primary production and decomposition processes in these habitats (Glińska‐Lewczuk 2009). Each aquatic ecosystem exhibits unique hydrological connectivity, resulting in exceptional habitat heterogeneity even over small river stretches, unlike other freshwater ecosystems. Limited connectivity or isolation from the river channel makes floodplain lakes specific ‘hot spots’ for biodiversity (Ward et al. 1999; Lew et al. 2016), but also hot spots for GHG emissions to the atmosphere. Wetlands, including floodplain lakes, are important and unpredictable sources of methane due to hydrological fluctuations, which influence the availability of substrates for methane production, its transport within the ecosystem and the responses of microbial community structure and activity, particularly among methanotrophs and methanogens (Hondula et al. 2021; Cui et al. 2024; Bechtold et al. 2025). Their contribution is significant, as wetlands account for approximately 20% of global CH_4_ emissions to the atmosphere (Saunois et al. 2020; Wang, Ge, et al. 2022; Wang, Li, et al. 2022) and are responsible for about 70% of methane emissions to freshwater systems (Sanches et al. 2019; Guggenheim et al. 2020). Bridgham et al. (2013) reported that wetlands are the single largest natural CH_4_ source with median emissions of 164 Tg year^−1^, which is about a third of total global emissions. However, there are considerable differences in emission rates from different types of wetlands. In natural lakes, CH_4_ flux generally occurs between 40% and 60% via ebullition (Bastviken et al. 2004, 2023). While CH_4_ emission rates are known to be controlled by a wide range of factors, including lake depth and sedimentation rates (Beaulieu et al. 2019), internal matter cycling, higher nutrient accumulation, more anoxic conditions and greater primary productivity generally lead to higher CO_2_ and CH_4_ fluxes. Nevertheless, microorganisms such as methanogens (methane producers, mGen) and methanotrophs (methane consumers, methanotrophic bacteria [MOB]) play a key role in regulating CH_4_ emissions to the atmosphere from riverine water bodies (Bastviken et al. 2023; Mustafa et al. 2024; Bechtold et al. 2025).

Methane is a GHG and the most stable carbon compound in anaerobic environments, playing a significant role as an intermediate in the mineralisation of organic matter (Kumar et al. 2021). However, estimates suggest that over 80% of CH_4_ produced in natural, undisturbed wetlands never reaches the atmosphere (Jensen et al. 2023). This is primarily due to a unique group of aerobic bacteria that can utilise CH_4_ as their sole source of energy and carbon (Hanson and Hanson 1996). Methanotrophs (MOB) act as microbial gatekeepers, serving as an effective biological filter that captures and oxidises CH_4_ before its release into the atmosphere (Jensen et al. 2023; Klomp et al. 2024).

In lakes and peatlands, the activity of methanogenesis and the structure of methanogenic microbiota depend on many factors including nutrient availability, habitat trophic level, physicochemical factors and their seasonal variability, as well as the quantity, composition and interactions of the microbiota, including methanotrophs (Klomp et al. 2024; Bastviken et al. 2023). Recent research on freshwater ecosystems has focused on understanding the effects of temperature, other environmental factors and the community composition and proportion of microorganisms involved in methane cycling on GHG emissions (Lew and Glińska‐Lewczuk 2018; Yang et al. 2020; Wang, Ge, et al. 2022; Wang, Li, et al. 2022).

The literature analysis conducted as part of this study reveals that methane production in freshwater ecosystems is more complex than previously understood, involving multiple pathways and contributing significantly to GHG emissions. Traditionally, it was believed that methane was produced exclusively in anoxic sediments through methanogenesis. However, recent research has demonstrated that methane can also be produced in the oxygenated upper layers (epilimnion) of lakes (Bižić‐Ionescu et al. 2018; Günthel et al. 2019; Guggenheim et al. 2020). Khatun et al. (2024) suggest that methane produced in the sublittoral zones can be transported over long distances (up to 20 km) into the metalimnion through processes like internal waves and tributary inflows. These mechanisms can induce sediment mixing, leading to the release of dissolved methane from the delta hypolimnion to the metalimnion.

Additionally, cyanobacteria, commonly found in freshwater ecosystems, have been identified as contributors to methane production under both light and dark, as well as oxic and anoxic conditions. Studies have shown that cyanobacteria can produce methane through the demethylation of methylphosphonates and by converting fixed inorganic carbon into methane (Tang et al. 2014, 2016; Visser et al. 2016; Bižic et al. 2020). Aquatic plants create favourable conditions for CH_4_ formation by providing extensive surfaces for microbial colonisation and abiotic methane production. Additionally, primary production in the water column supplies labile organic matter that fuels both aerobic and anaerobic CH_4_ formation, as demonstrated by studies of freshwater ecosystems (Grasset et al. 2018; Kuhn et al. 2021; Bastviken et al. 2023).

Such interactions between aquatic primary producers, organic carbon availability and methane formation highlight the central role of microbial communities in regulating GHG fluxes. In particular, MOB and methanogenic archaea (mGen) are key drivers of methane cycling in freshwater ecosystems. However, little is known about the role of these microorganisms in GHG emissions from floodplain lakes, which represent diverse, successively changing ecosystems characterised by variable oxygenation levels, nutrient content and trophic status (Cui et al. 2024; Mustafa et al. 2024). The condition of these ecosystems directly influences the magnitude of GHG emissions to the atmosphere. Successional changes in floodplain ecosystems may create particularly favourable conditions for the formation and emission of GHG. However, insufficient knowledge in this area highlights the need for further studies of these habitats and the development of strategies to mitigate their contribution to global warming.

This study aims to explore the relationships between CH_4_ emissions to the atmosphere and the community composition of methanogenic archaea and MOB, as well as their interactions with various physicochemical parameters in floodplain lakes at different successional stages. Since global warming triggers feedback loops that further increase temperatures in freshwater ecosystems, we sought to determine how these changes may affect methane fluxes to the atmosphere. Specifically, we investigated how microbial communities responsible for methane oxidation and production in natural floodplain lakes respond to these changes. To achieve our objectives, we tested the following hypotheses: (i) in early successional oxbow lakes, even a small presence of MOB under conditions favourable for methanogenesis can significantly limit methane emissions to the atmosphere; (ii) habitat conditions of oxbow lakes and their hydrological connectivity with the river influence seasonal fluctuations in the proportion of methanogens to methanotrophs; (iii) while water temperature is an important factor regulating the microbial community involved in methane cycling, it is not the primary driver of increased methane flux to the atmosphere.

## 2 Materials and Methods

### 2.1 Study Sites

**FIGURE 1:**
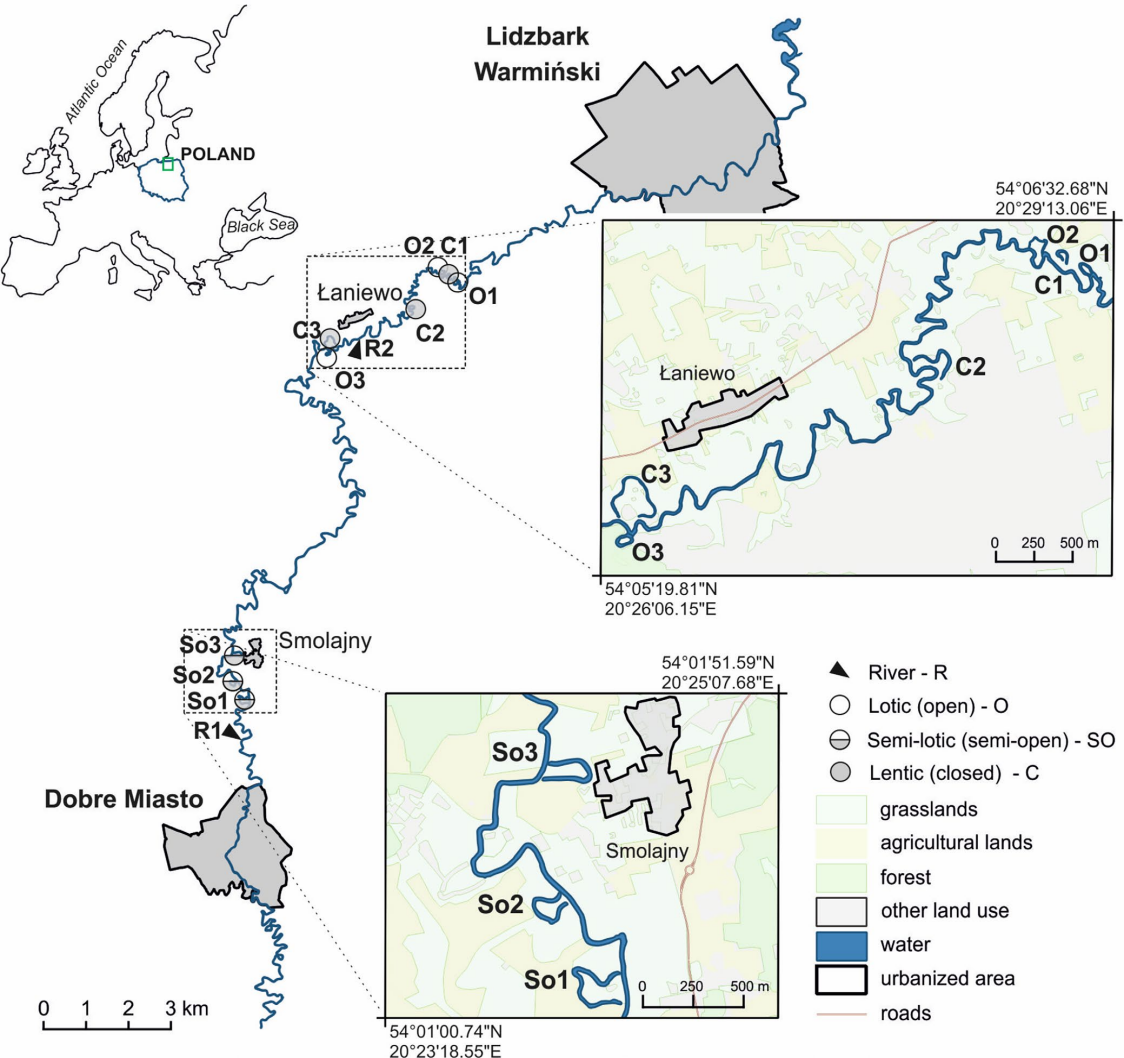
Location of the study area and studied objects (floodplain lakes) along the Łyna River in its middle course.

This study was conducted in the free‐flowing section of the Łyna River, located in north‐eastern Poland (Figure 1). At the village of Smolajny, the river drains a catchment area of 2290 km^2^. The average river flow is 14 m^3^/s, peaking at 35 m^3^/s during flood events. Flooding typically occurs when water flow exceeds 22 m^3^/s, with a re‐occurrence period of 1.4 years calculated over a 40‐year cycle (Glińska‐Lewczuk 2005). The river tends to meander in this section, and floodplain lakes show various stages of evolution and maturity. Each cut‐off is elongated with a single‐loop form inherited from the former river channel. Their lengths range from 200 to 400 m, with an average sinuosity of 3.0. Surface areas are relatively small, approximately 1 ha, and maximum depths are ca. 2.5 m. The coverage of oxbow lakes by aquatic plants varies significantly, from 0.11% in lotic oxbows to 56% in lentic oxbows. The number of aquatic taxa per oxbow ranges from 3 to 11, including submerged species such as *Potamogeton* spp., *Ceratophyllum demersum* and *Elodea canadensis* . Floating‐leaved plants like *Nuphar lutea* and *Nymphaea alba* dominate the water surface, forming dense mats in semi‐lotic and lentic oxbows. Emergent vegetation, including *Phragmites australis* and *Typha* spp., is abundant along the margins of the lakes. This vegetation contributes to sediment stabilisation and provides shelter and nesting sites for various aquatic and terrestrial species. These plant communities play a crucial role in maintaining ecological balance, supporting biodiversity and enhancing the ecosystem health of the Łyna River floodplain ecosystems. A detailed description of the study sites and the area can be found in the works of Glińska‐Lewczuk (2005, 2009).

For the purpose of this study, 11 study sites were selected (Figure 1). The floodplain lakes were classified into three groups with respect to the degree of hydrological connectivity: lotic, which maintain continuous connection with both arms of the river channel; semi‐lotic (also referred to as semi‐open), which are connected to the river via a single downstream arm; and lentic (closed), which are hydrologically isolated from the river except during flood events. In lotic and semi‐lotic types, the ongoing exchange of water and matter with the river supports aeration and limits the accumulation of organic material. In contrast, isolated lentic oxbows are dominated by internal matter cycling and exhibit long water retention times. These conditions, coupled with advanced macrophyte succession and thick sediment layers, promote organic matter accumulation, overgrowth and silting (Kobus et al. 2016). Their tendency to accumulate organic matter promotes overgrowing and silting‐up, which is the highest compared to lotic and semi‐lotic types. As a result, each type has a higher potential for methane production and emission due to the prevalence of anoxic conditions and rich organic substrates, compared to their lotic and semi‐lotic counterparts. This differentiation in methane production and emission potential among oxbow types—driven by variation in oxic conditions and organic substrate availability—is of particular relevance to the objectives of this study, as it highlights the role of hydrological connectivity in regulating GHG dynamics in floodplain ecosystems.

### 2.2 Water Sampling and Analytical Procedure

For this study, floodplain lakes of the Łyna River were monitored from January 2021 to December 2022 on a seasonal basis, with four sampling campaigns conducted each year: in winter (December or January), spring (May), summer (August), and autumn (October). Biological sampling and hydrochemical data were collected from three locations on each lake: upstream, downstream and the middle part, the most distant part from the river channel. In total, 384 water samples were collected, including samples taken from the river at two sites, R1 and R2. Water temperature (°C), pH, dissolved oxygen (DO, mg L^−1^), turbidity (NTU), electrical conductivity (EC, μS cm^−1^) and chlorophyll‐*a* (Chl–*a*, μg L^−1^) were measured using the YSI 6600R2 multiprobe (Yellow Springs, OH, USA) after calibration. The physical properties of oxbow lake water were measured at a depth of approximately 0.3 m below the water surface.

Water samples for laboratory analyses were collected in 2.5‐L polyethylene bottles, kept cold and in darkness until analysed within 12 h. These samples were analysed for total nitrogen (TN, mg L^−1^) using a total organic carbon (TOC) analyser (multi N/C 3100 TOC/TNb Analyser, Analytik Jena GmbH+Co. KG, Germany). Chemical oxygen demand (COD) was determined using the dichromate closed reflux method. The concentrations of nitrates (NO_3_–N), ammonia (NH_4_–N) and orthophosphates (PO_4_–P) in water samples were determined using ion chromatography (IC) with a DIONEX ICS‐5000 system (Thermo Fisher Scientific), following filtration through 0.45 μm membrane filters to remove suspended solids. Total phosphorus (TP, mg L^−1^) was measured using the ascorbic acid colorimetric method following potassium persulfate digestion, in accordance with the APHA 2017.

Carbon dioxide (CO_2_) and methane (CH_4_) emissions (hereafter eCO_2_ and eCH_4_, respectively) were measured concurrently with the microbiological sampling and physicochemical measurements of water, using the floating chamber technique. A portable CRDS laser analyser (GasScouter G4301, Picarro Inc., Santa Clara, CA, USA) coupled with a portable cylindrical chamber (volume 0.004 m^3^, water‐air interface area 0.0615 m^2^) was used. The floating chamber method captures the net emission rate of gases from the water to the air, accounting for both molecular diffusion and any micro‐ and large bubbles that entered the chamber. The flux calculation involved measuring changes in gas concentration over time (Δ*C*/Δ*t*) at an average frequency of 72 readings per minute. Each measurement, taken during midday hours, lasted 3 minu to allow for proper gas mixing at a sample flow rate of approximately 1 L/min at atmospheric pressure and accurate recording of non‐steady state fluxes (Hu et al. 2014). Measurements were performed in triplicate. The flux rates (eCH_4_ in mg m^−2^ day^−1^, eCO_2_ in g m^−2^ day^−1^) were determined using the following equation (Skwierawski 2024):

$${\mathrm{eCH}}_{4},{\mathrm{eCO}}_{2}=\frac{\;\left(C_{180}-C_{0}\right)\cdot \rho \cdot 0.004\cdot 480}{0.0615}$$

where *C*_0_ is the gas concentration in the air in the measurement system at the beginning of the measurement (ppmv); *C*_180_ is the gas concentration in the air in the measurement system after 180 s of measurement (ppmv); *ρ* is the volumetric density of methane or carbon dioxide (kg/m^3^); 0.004 is the volume of the closed measurement system (m^3^); 480 is the time conversion factor from 3 min to a day; 0.0615 is the surface area of the water surface covered by the measurement (m^2^).

### 2.3 Microbial Procedure

#### 2.3.1 Total Microbial Number

To determine the total microbial number (TMN), the technique of direct microbial counting on a filter with epifluorescence microscopy, as described by Porter and Feig (1980), was utilised. Triplicate subsamples were fixed with neutralised formaldehyde (pH 7.4; final concentration 4%) and stored at 4°C until analysis. Subsamples were stained with 4′,6‐diamidino‐2‐phenylindole (DAPI; final concentration 0.01 g mL^−1^) for 15 min in the dark. Subsequently, the samples were gently filtered through 0.2‐μm black Nuclepore filters (type GTTP, Millipore) and enumerated under an Olympus epifluorescence microscope, with more than 1000 bacterial cells per sample.

#### 2.3.2 DOPE‐FISH

For DOPE‐FISH analysis, 50 mL samples were fixed with paraformaldehyde (4% final concentration, adjusted to pH 7.4). After fixation, samples were filtered onto white polycarbonate filters (Millipore, Type GTTP, 0.2 μm pore size, 47 mm diameter) and stored at −20°C until further processing. The DOPE‐FISH procedure was conducted as described by Glöckner et al. (1996) and Pernthaler et al. (2001), with modifications by Stoecker et al. (2010). For identifying aerobic methane‐oxidising bacteria (MOB), probes M84 (for MOB_I_, *Methylococcaceae*) and M450 (for MOB_II_, *Methylocystaceae*) were used (Eller et al. 2001). The probe Arch915 was used to determine the presence of *Archaea*, including *Crenarchaeota*, while *Euryarchaeota* (Eury806), which includes methanogens, was identified using the probes described by Stahl and Amann (1991). Methanogenic *Archaea* were identified using three different oligonucleotide probes: MB311 for *Methanobacteriales* (mGen_I_), MSMX86 for *Methanosarcinales* (mGen_II_) and MGen1200b for most *Methanomicrobiales* (mGen_III_) (Crocetti et al. 2006). The characteristic oligonucleotide probes used in this study are listed in Table 1. Autofluorescence and nonspecifically stained cells were determined with the NON338 negative control probe (Wallner et al. 1993). All oligonucleotide probes were double‐labelled with the Cy3 dye (Stoecker et al. 2010). Cells on the filter sections were observed using an epifluorescence microscope equipped with filter sets for DAPI and Cy3. The fractions of DOPE‐FISH stained cells were quantified in triplicate, with at least 1000 DAPI‐stained cells per sample.

**TABLE 1:** Characteristic oligonucleotide probes used in this study.

Probe	Sequence	FA (%)^a^	Specificity	References
Mg84 MOBI	5′‐CCA CTC GTC AGC GCC CGA‐3′	20	*Methylococcaceae*	Eller et al. (2001)
Ma450 MOBII	5′‐ATC CAG GTA CCG TCA TTA TC‐3′	20	*Methylocystaceae*	Eller et al. (2001)
Arch915	5′‐GTG CTC CCC CGC CAA TTC CT‐3′	20	*Archaea*	Loy et al. (2002)
Eury806	5′‐CAC AGC GTT TAC ACC TAG‐3′	20	*Euryarchaea*	Teira et al. (2004)
MB311	5′‐ACC TTG TCT CAG GTT CCA TCT CC‐3′	30	*Methanobacteriales*	Crocetti et al. (2006)
MGen1200b	5′‐CGG ATA ATT CGG GGC ATG CTG‐3′	20	*Methanomicrobiales*	Crocetti et al. (2006)
MSMX860	5′‐GGC TCG CTT CAC GGC TTC CCT‐3′	45	*Methanosarcinales*	Raskin et al. (1994)

^a^ Formamide FA [%]: formamide concentration in the hybridization buffer to ensure specific detection of target organisms.

#### 2.3.3 MOB/mGen Ratio

Understanding the relationship between methane dynamics and microbial activity is essential for assessing the balance between CH_4_ production and oxidation, and for identifying conditions that support microbial control of methane emissions (Conrad 2009; Rey‐Sanchez et al. 2019; Hanson and Hanson 1996). To assess the balance between methane production and its microbial oxidation across aquatic ecosystems of varying connectivity, we used the ratio of methane‐oxidising bacteria to methanogens (MOB/mGen) in combination with measured methane fluxes (eCH_4_). Samples were assigned to water body types based on site hydrology (river, lotic, semi‐lotic or lentic oxbows), and categorised into four functional classes centered around the equilibrium value of 1. Class II represents balanced communities (0.8–2.0), while Class I (> 2.0) indicates methanotroph dominance and Classes III (0.4–0.8) and IV (< 0.4) reflect increasing methanogen prevalence. Class zones were annotated directly, and a power‐law regression was applied to the full dataset, yielding a fitted equation and coefficient of determination (*R* ^2^) to quantify the strength of the inverse relationship between methane emission and methanotroph dominance.

### 2.4 Statistical Analyses

We performed analysis of variance tests (two‐way ANOVA followed by Duncan's multiple range test (MRT) as a post hoc procedure at *p* ≤ 0.05) to evaluate abundances of microbial groups among different types of floodplain water bodies and across seasons, using lake type and season as explanatory variables. Hydrochemical parameters were compared between sampling sites using a one‐way analysis of variance (ANOVA).

To assess the impact of environmental factors on the microbial community in the studied oxbows and the Łyna River, partial least squares regression (PLS‐R) was employed. PLS‐R, a multivariate statistical technique combining elements from principal component analysis and multiple regression, is particularly suited for ecological studies with numerous and highly collinear predictor variables (Shawul et al. 2019). This technique enabled us to identify the most influential factors affecting methanotrophs and methanogens. The methanotrophs analysed included *Methylococcaceae* (MOB_I_ probe: Mg84) and *Methylocystaceae* (MOB_II_ probe: Ma450), while the methanogens included *Methanobacteriales* (mGen_I_ probe: MB311), *Methanosarcinales* (mGen_II_ probe: MSMX860) and *Methanomicrobiales* (mGen_III_ probe: mGen1200b), as well as *Archaea* (probe: Arch915) and *Euryarcheota* (probe: Eury806) as response (Y) variables. Environmental qualitative variables (water quality) and quantitative variables (oxbow types) were used as explanatory variables (*X*). The most significant predictors were identified by the variable importance in projection (VIP) scores in the PLS‐R model. According to Wold et al. (2001), predictors (*X*) with VIP scores > 1 are considered more important than average in explaining the variance in the response variables across all components of the model.

To refine the selection of key predictors in the PLS‐R model, we applied the variable identification (VID) technique. VID scores indicate the strength of a variable's influence on the response variable. Positive VID scores denote positive associations, while negative scores denote adverse relationships. A VID score cut‐off at 0.4 was used to indicate significant variables, provided the confidence interval for the standardised coefficient of a given predictor did not include zero. The robustness of the PLS‐R model was evaluated through diagnostic checks, including RMSE, MSE and the examination of the predictive relevance (*Q* ^2^) of the model. The PLS‐R analysis was performed using XLSTAT software by Lumiero, an MS Excel add‐in statistical tool (www.xlstat.com).

The Generalised Chi‐squared Automatic Interaction Detector (GCHAID) tree analysis was utilised to explore the relationships between eCH_4_ from different types of floodplain lakes and the MOB/mGen ratio. The GCHAID algorithm is suitable for handling various types of response variables, including continuous variables like CH_4_ emission. The primary splitting criterion is based on the chi‐squared (*χ* ^2^) test, identifying the most significant predictor variable at each node. Splitting continued until one of the following stopping criteria was met: a minimum number of cases in a node (95), maximum tree depth defined as 3, and a significance level for the *χ* ^2^‐test set at *p* ≤ 0.05. The GCHAID tree construction was performed using Statistica ver. 13.3 (TIBCO Inc., Tulsa USA).

## 3 Results

### 3.1 Physicochemical Properties of Floodplain Lakes Water in Relation to Hydrological Connectivity Gradient

The water quality data in Table 2 provide a comprehensive overview of the physical and chemical properties of the studied lakes. The water exhibits near‐neutral pH and an average degree of mineralisation. Water temperature follows a typical seasonal pattern for the temperate climatic zone, with minimum values of > 0.3°C in January and February, and maximum values of > 20°C in July. The temperature readings indicate that all water bodies share a similar thermal regime with average water temperatures comparable among oxbows, showing no significant differences (*p* ≤ 0.05). Lotic, semi‐lotic and lentic ecosystems, categorised based on their hydrological connectivity with the river channel, exhibit significant differences in dissolved oxygen, chlorophyll‐*a* and nutrient concentrations, which have implications for CO_2_ and CH_4_ emission dynamics.

**TABLE 2:** Summary of the physical and chemical properties of water in the Łyna River and oxbow lakes (mean ± SD) from January 2021 to December 2022.

Parameter	Unit	River (*N* = 24)		Lotic (*N* = 108)		Semi‐lotic (*N* = 144)		Lentic (*N* = 108)		Total (*N* = 384)	
		Mean	±SD	Mean	±SD	Mean	±SD	Mean	±SD	Mean	±SD
pH	—	7.76	0.32	7.55	0.40	7.69	0.37	7.57	0.47	7.62	0.41
Temperature	°C	10.19	6.32	10.53	5.96	10.79	7.48	10.05	7.69	10.47	7.06
DO	mg L^−1^	6.03	0.97	6.15	1.11	5.32	2.05	4.10	2.10	5.26	1.96
Chl–*a*	μg L^−1^	6.28^ab^	5.07	4.26^a^	1.75	8.80^bc^	8.65	11.18^c^	8.23	8.04	7.52
EC	μS cm^−1^	441	23.4	456	25.6	449	34.5	458	188	453	103.2
NO_3_–N	m L^−1^	0.70^b^	0.13	0.73^b^	0.54	0.36^a^	0.25	0.39^a^	0.74	0.49	0.54
NH_4_–N	mg L^−1^	0.53^b^	0.27	0.34^a^	0.28	0.49^b^	0.44	0.58^b^	0.45	0.47	0.40
PO_4_–P	mg L^−1^	0.17^a^	0.02	0.16^a^	0.05	0.17^a^	0.11	0.28^b^	0.16	0.20	0.12
TP	mg L^−1^	0.37^a^	0.06	0.36^a^	0.10	0.34^a^	0.18	0.88^b^	0.60	0.50	0.41
COD	mg L^−1^	23.92^a^	1.91	32.73^b^	6.91	26.02^a^	5.73	40.74^c^	16.22	31.92	11.77
Turbidity	NTU	3.29^a^	1.40	3.97^a^	1.54	7.29^b^	6.59	6.69^b^	7.60	5.94	5.96
eCO_2_	g m^2^ day^−1^	0.65^ab^	0.41	0.77^b^	0.46	0.61^ab^	0.37	0.99^c^	0.31	0.76	0.42
eCH_4_	mg m^2^ day^−1^	32.06^a^	33.49	21.68^a^	13.59	225.42^b^	267.09	506.91^c^	317.68	235.20	302.07

*Note:* Different superscripts denote significant differences between floodplain lake types (one‐way ANOVA, Duncan post hoc test, *p* ≤ 0.05).

Lotic oxbow lakes had the highest oxygen concentrations (mean 6.15 ± 1.11 mg L^−1^) compared to semi‐lotic (mean 5.32 ± 2.05 mg L^−1^) and lentic ecosystems (mean 4.10 ± 2.10 mg L^−1^). Lotic environments benefit from stable oxygenation due to the continuous water exchange with the river which replenishes oxygen levels and leads to lower GHG production. In lotic lakes, Chl‐*a* concentrations were significantly lower (mean 4.26 ± 1.75 μg L^−1^), indicating lower primary productivity and nutrient accumulation compared to more isolated water bodies. Semi‐lotic lakes were characterised by average 8.80 ± 8.65 μg Chl‐*a* L^−1^, while lentic by 11.18 ± 8.23 μg Chl‐*a* L^−1^.

Lentic floodplain lakes are the most fertile environments rich in nutrients, such as TP and phosphate‐phosphorus (PO_4_–P) (0.88 ± 0.60 and 0.28 ± 0.16 mg L^−1^, respectively), and exhibit the highest COD values (40.74 ± 16.22 mg L^−1^). The EC in lentic oxbow lakes was the highest (458 ± 188 μS cm^−1^), though not significantly different from other lakes.

A marked seasonal pattern in TP concentrations was observed, with maximum levels during summer when internal phosphorus cycling at high water temperatures and oxygen deficits at the bottom occur (see also Glińska‐Lewczuk et al. 2009). Nitrate‐nitrogen levels were higher in open oxbow lakes (mean 0.73 ± 0.54 mg L^−1^), indicative of allochthonous nutrient input from the river or reclamation ditches. On the other hand, lower NO_3_–N levels in oxbow lakes with limited water exchange and abundant macrophytes results from high bioaccumulation rates and denitrification processes during warm seasons. Conversely, during winter, NO_3_–N concentrations increased, as uptake by phytoplankton and denitrifying bacteria is minimal. Ammonium‐nitrogen concentrations are lower in water of lotic oxbows (mean 0.34 ± 0.28 mg L^−1^) due to better oxygenation. Significantly higher NH_4_–N levels in lentic oxbows (mean 0.58 ± 0.45 mg L^−1^) are elevated by anaerobic decomposition in summer and autumn.

The data reveal significant variations in eCO_2_ and eCH_4_ from different water bodies, particularly among rivers and various types of floodplain lakes (Table 2). The mean annual values of carbon dioxide emissions were 0.65 ± 0.41 for rivers, 0.77 ± 0.46 for lotic lakes, 0.61 ± 0.37 for semi‐open lakes and 0.99 ± 0.31 for isolated lakes. Notably, lake isolation from the river channel was associated with elevated methane emissions. Methane fluxes in lentic lakes (mean value of 506.91 ± 317.68) were significantly higher (ANOVA, Duncan test, *p* ≤ 0.05) than those observed in rivers (mean 32.06 ± 33.49) and in ecosystems connected to the river channel—21.68 ± 13.59 in lotic and 225.42 ± 267.09 in semi‐lotic lakes.

### 3.2 MOB and mGen in the Microbiome Community

**FIGURE 2:**
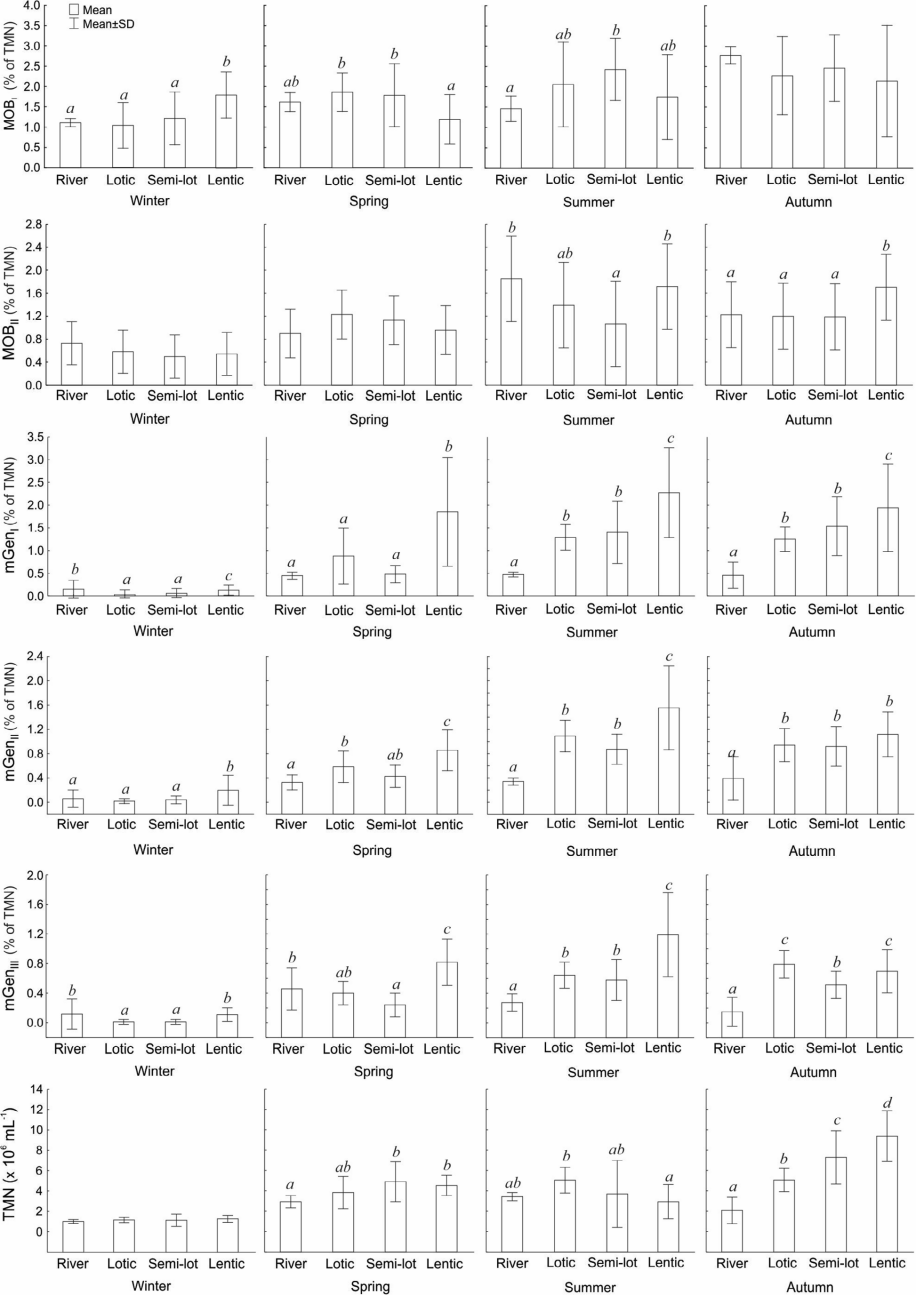
Seasonal structure of methanotrophs, methanogens, and total microbial number (TMN) in relation to different types of floodplain lakes and the Łyna River. Significant differences, indicated by superscripts, were determined using the Duncan post hoc test following one‐way ANOVA (*p* ≤ 0.05).

The studied sites exhibited significant variation in microbial abundance, both among habitat types and across seasons. In the Łyna River, TMNs were generally the lowest compared to oxbow lakes, with mean values ranging from 1.01 to 3.43 × 10^6^ mL^−1^ (Figure 2). Methane‐oxidising bacteria (MOB_I+II_) were present throughout the study season, comprising from 1.84% to 4.00% of the microbial community. During summer, the river showed a significantly higher proportion of MOB_II_ (ANOVA, Duncan post hoc test, *p* ≤ 0.05), averaging 1.85%. Methanogens were also detected year‐round, but their relative abundance was consistently lower than MOB_I+II_, ranging from 0.33% to 1.24%. The relative abundance of *Archaea* and *Euryarcheota* did not differ significantly between the river and oxbow lakes.

In lotic lakes, the TMN ranged from 1.16 to 5.06 10^6^ mL^−1^. The abundance of MOB_I_ and MOB_II_ did not differ significantly across seasons compared to their abundance in the river. Although MOB_I+II_ constituted a relatively small proportion of the microbial community throughout the year, their abundance showed an increasing trend from winter (1.62%) to autumn (3.47%). Methanogen abundance followed a similar seasonal pattern, peaking in summer with an average of 3.03 × 10^6^ mL^−1^.

In semi‐lotic lakes, TMN ranged from 1.13 to 7.29 × 10^6^ mL^−1^. Methanotrophs and methanogens accounted for 2.93% and 1.78% of TMN, respectively. The seasonal dynamics of MOB_I+II_ and mGen in semi‐lotic oxbows were more similar to those in lotic oxbow lakes than in isolated ones, as c supported by significant correlations between their relative abundances and TMN (*r* = 0.488, *p* ≤ 0.001 and *r* = 0.639, *p* ≤ 0.001, respectively).

In lentic oxbow lakes, TMN averaged 4.53 × 10^6^ mL^−1^, peaking in autumn (average 9.37 × 10^6^ mL^−1^), which significantly distinguished them from lakes connected with the river (*p* ≤ 0.05, Figure 2). Methanotrophs accounted for an average of 2.95% of TMN. MOB_I_ was most abundant in autumn and winter, while MOB_II_ was significantly more numerous in summer and autumn (ANOVA, Duncan test, *p* ≤ 0.05), comprising 1.72% and 1.23% of the microbial community, respectively. In general, spring and summer were less favourable for methanotroph development in isolated oxbows. Methanogens constituted an average of 5.02% of TMN, with the highest relative abundance (> 9%) observed in summer, and elevated levels maintained from spring to autumn (ANOVA, Duncan test, *p* ≤ 0.05). *Methanobacteriales* (mGen_I_) dominated across all seasons and sites, reaching peak densities in isolated oxbows during summer. The less abundant groups—*Methanosarcinales* (mGen_II_) and *Methanomicrobiales* (mGen_III_) also peaked in isolated lakes during summer and autumn, with higher spring abundances in these habitats compared to other oxbow types. All methanogen groups were least abundant in winter.

To assess the balance between methane production and its oxidation under varying environmental conditions in the studied oxbow lakes, we calculated the ratio of methanotrophs to methanogens (MOB/mGen ratio). The highest values of this ratio, classified as Class I (MOB/mGen > 2.0) and indicative of methanotroph dominance, were observed primarily during winter, particularly in the river and lotic oxbow lakes. In Class I samples, where methanotrophs dominated the methane‐cycling community, CH_4_ emissions remained low, not exceeding 30 mg m^−2^ day^−1^, highlighting their role as effective ‘gatekeepers’ of methane flux. Class II, defined by a balanced ratio of MOB (I and II) to methanogens (I–III), was identified in lotic oxbow lakes during spring to autumn, and in semi‐lotic lakes during summer and autumn. This class reflected a seasonal microbial steady state, with methane fluxes generally not exceeding 300 mg·m^−2^·day^−1^. In contrast, Classes III and IV (MOB/mGen ratios of 0.4–0.8 and < 0.4, respectively), where methanogens were dominant, were characteristic of stagnant waters in isolated oxbow lakes, particularly from spring through autumn. Under conditions of elevated temperature, abundant organic matter and limited water exchange, these habitats exhibited high CH_4_ emissions, often exceeding 750 mg m^−2^·day^−1^. The lowest MOB/mGen ratios clearly reflected methanogen dominance. Class IV, in particular, represents a state of pronounced methanogenic activity, indicating that the ecosystem is highly prone to methane production with minimal microbial oxidation.

**FIGURE 3:**
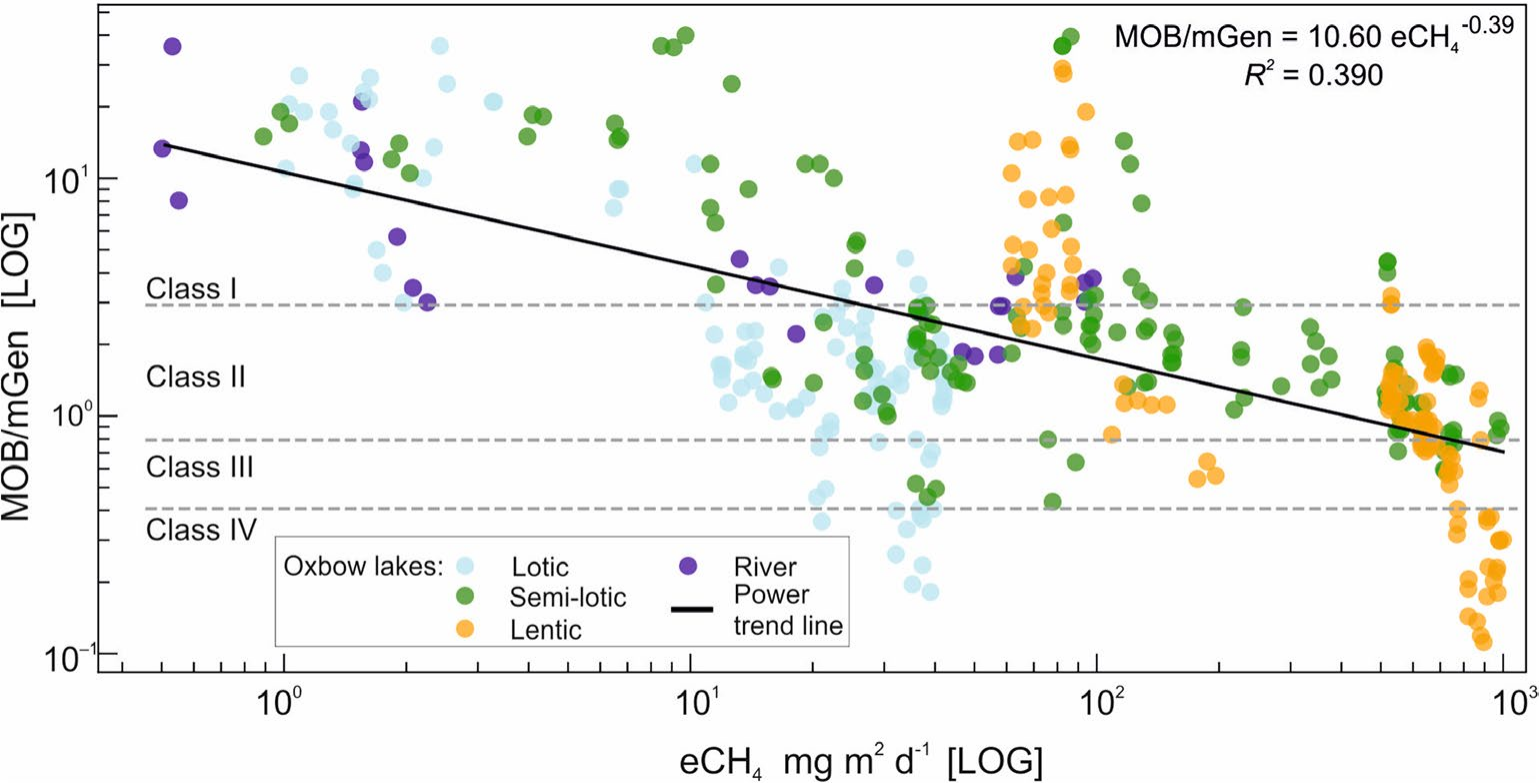
Regression between the average MOB/mGen ratio and CH_4_ emission (mg m^−2^ day^−1^) for oxbow lake types and the Łyna River, with a trend line and functional class boundaries.

A regression analysis revealed a significant inverse relationship between the MOB/mGen ratio and CH_4_ emissions (eCH_4_), with a determination coefficient of *R* ^2^ = 0.39, indicating that lower MOB/mGen ratios were consistently associated with higher CH_4_ fluxes (Figure 3).

Summary of the classification based on the MOB/mGen ratio is presented in Table 3, outlining four microbial community types that reflect varying balances between methane oxidation and production. Each class is linked to specific water body types and seasonal contexts, highlighting the ecological conditions under which microbial control of methane flux is either maintained or diminished.

**TABLE 3:** Classification of microbial methane‐cycling communities based on MOB/mGen ratio with reference to water body type and seasonality.

Class	MOB/mGen range	Dominant type of water body	Dominant seasons	CH_4_ flux^a^ mean range (mg·m^−2^·day^−1^)	Description
Class 1	> 2.0	River, bi‐connected oxbows, well‐aerated, flowing water	Winter, spring	< 30	Strong MOB dominance; high oxidation capacity. Strong microbial CH_4_ sink. In winter may characterise semi‐lotic or lentic oxbows
Class II	0.8–2.0	Lotic and semi‐lotic oxbows supplied with river water	Spring, autumn	30–300	Balanced community; near‐equilibrium conditions. Moderate CH_4_ flux; strong microbial cycling, especially in summer
Class III	0.4–0.8	Semi‐lotic or lentic oxbows with stagnant water	Spring, autumn	300–750	Mitigated lentic systems with persistent CH_4_ emission but partial microbial control. Moderate methanogen dominance; increasing CH_4_ flux
Class IV	< 0.4	Lentic oxbows with stagnant water abundant in organic matter	Summer, early autumn	> 750	Emission‐prone lentic habitats; limited MOB presence and high CH_4_ output, especially in summer. Strong methanogen dominance; high CH_4_ production

^a^ Based on the regression trend in Figure 3.

**FIGURE 4:**
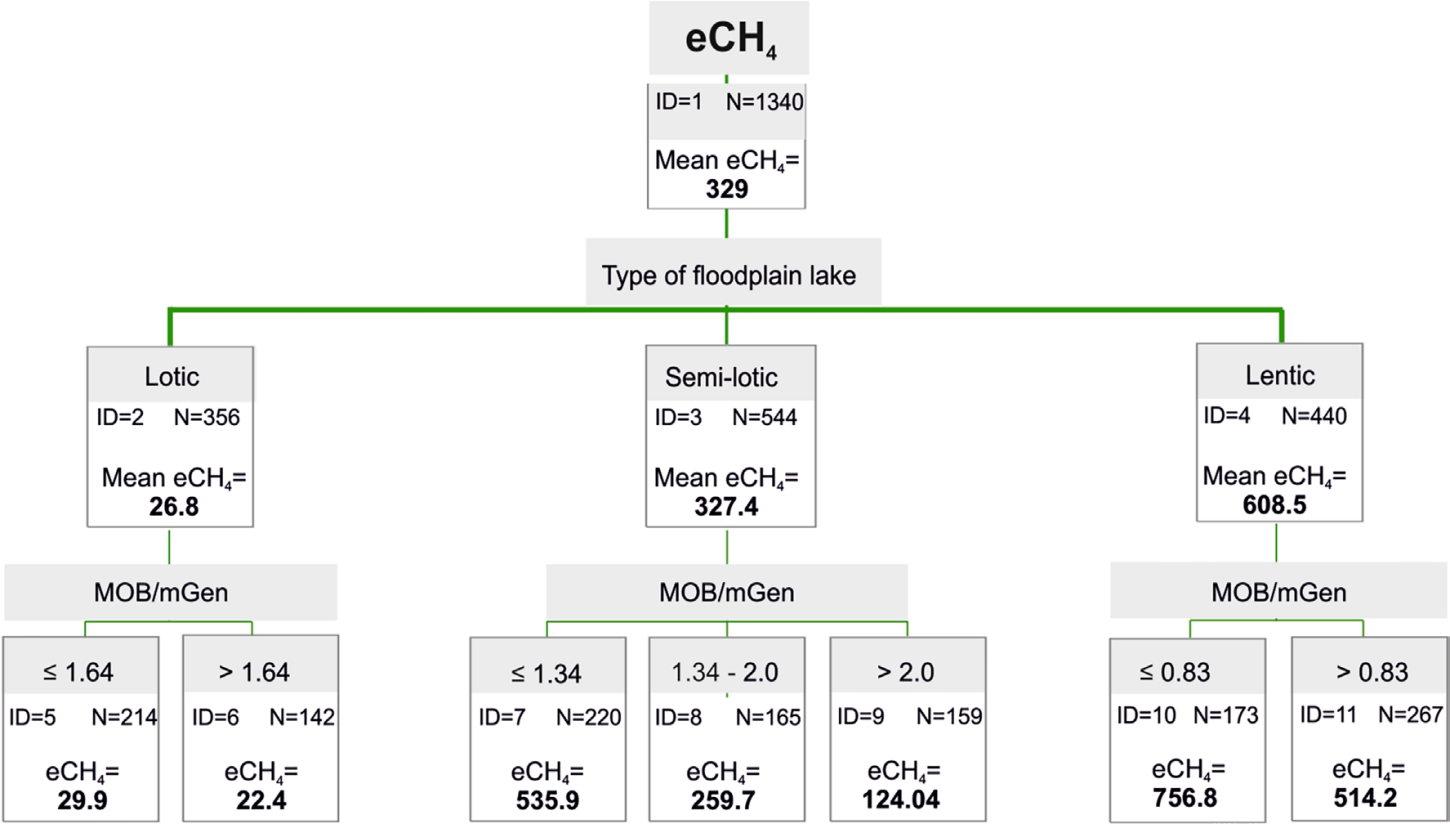
GCHAID Decision Tree Analysis of CH_4_ emissions from floodplain lakes (lotic, semi‐lotic and lentic) based on the MOB/mGen ratio. Each node displays the number of observations (N) and mean CH_4_ emission (mean CH_4_ in mg·m^−2^·day^−1^). The splits highlight the significant factors influencing CH_4_ emissions with lower MOB/mGen values. The tree identifies 4 split nodes and 7 terminal nodes, providing a detailed breakdown of how these variables interact to affect CH_4_ emission levels.

The GCHAID tree (Figure 4) confirmed a clear gradient of eCH_4_ inversely related to MOB/mGen ratio values across the lake types. The first‐level split, by lake type, demonstrates that hydrological connectivity is the primary determinant of methane emissions, with lentic systems showing the highest mean eCH_4_ (608.5 mg·m^−2^·day^−1^) and lotic systems the lowest (26.8 mg·m^−2^·day^−1^). The second‐level splits reveal strong modulation by the MOB/mGen ratio, confirming that microbial composition further stratifies emission potential, even within the same lake type. The analysis indicates that the MOB/mGen ratio is a reliable predictor of methane emissions across all oxbow types, with lower ratios consistently associated with higher eCH_4_ fluxes.

### 3.3 PLS‐R Analysis

**FIGURE 5:**
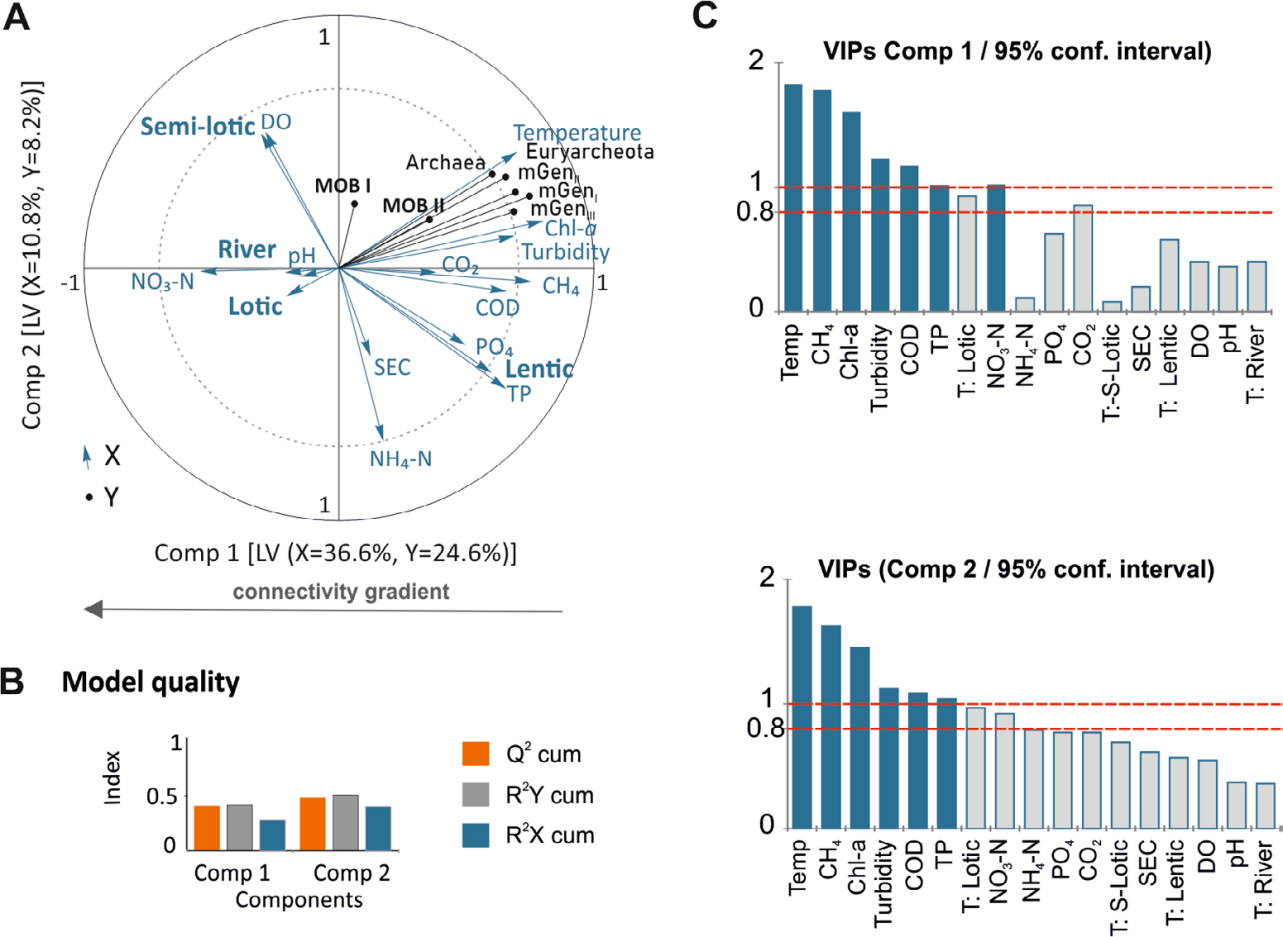
Partial least squares (PLS) regression biplot reflecting the effect of water quality parameters (quantitative variables) and types of floodplain lakes (qualitative variables) as the explanatory variables (*X*) on the microbial groups (*Y*). Inner dashed circle denotes correlation coefficient with *r* = 0.75 (A). PLS‐R model quality (B). VIPs (variable influence on projection) for each explanatory variable of Component 1 and Component 2. VIP diagrams show relative importance of predictors. VIPs > 0.8, based on Wold's criteria (Wold et al. 2001), indicate that the predictor variable is considered to be significantly important to the corresponding dependent variable (C).

The PLS‐R analysis (Figure 5A–C) illustrates the relationships between environmental variables and the distribution of methanotrophs (MOB_I_ and MOB_II_), methanogens (represented by vectors such as mGen_I‐III_), Archaea and Euryarchaeota across semi‐lotic, lotic and lentic oxbow lake types, as well as the river. In Figure 5A, the PLS biplot shows that microbial community distribution aligns with the gradient of hydrological connectivity, reflecting distinct ecological niches and responses to environmental conditions. Methanotrophs and methanogens were associated with key environmental parameters, such as water temperature, CH_4_, Chl‐*a*, turbidity, COD and TP. VIP scores > 0.8 (Figure 5C) further highlight these variables as significant predictors in both PLS components.

The VID procedure as part of PLS‐R revealed that out of 17 environmental explanatory variables, water temperature is the most significant factor positively influencing the presence of methane‐related bacterial groups in the studied water bodies (Table 4). VID scores for temperature exceeded 0.8 for methane‐oxidising bacteria MOB_II_ (VID = 0.819), *Archaea* and *Euryarchaeota* (VID = 0.818 and VID = 0.816, respectively), as well as *Methanosarcinales* and *Methanomicrobiales* (VID = 0.806 and VID = 0.803, respectively). Other variables that positively impacted these bacteria include Chl‐*a* (VID from 0.776 to 0.799), water turbidity (VID from 0.636 to 0.672), CH_4_ (VID from 0.599 to 0.677) and COD (VID from 0.499 to 0.569). These factors favoured lentic conditions, particularly for MOB_II_ and methanogens (*Methanobacteriales* and *Methanomicrobiales*). TP was also identified as an important predictor for methanogens.

**TABLE 4:** Discriminative components for each bacterial group selected through the variable identification (VID) procedure (PLS‐R) and listed in the decreasing order of VID score.

Parameter	MOB_I_ (Ma450%)		MOB_II_ (Mg84%)		*Archaea* (Arch915%)		*Euryarcheota* (Eury806%)		Methanogens mGen_I‐III_		*Methanobacteriales* mGen_I_ (MB311%)		*Methanosarcinales* mGen_II_ (MSMX860%)		Methanomicrobiales mGen_III_ (MG1200b%)	
R^2^	0.068		0.163		0.501		0.559		0.640		0.564		0.569		0.523	
Std. deviation	0.948		0.640		2.125		1.340		1.105		0.656		0.372		0.300	
MSE	0.890		0.406		4.478		1.779		1.210		0.426		0.137		0.089	
RMSE	0.943		0.637		2.116		1.334		1.100		0.653		0.370		0.299	
	**X**	**VID**	**X**	**VID**	**X**	**VID**	**X**	**VID**	**X**	**VID**	**X**	**VID**	**X**	**VID**	**X**	**VID**
Positive	Temp.	0.599	Temp.	0.819	Temp.	0.818	Temp.	0.816	Temp.	0.798	Temp.	0.798	Temp.	0.806	Chl‐*a*	0.803
	T: Lotic	0.441	Chl‐*a*	0.778	Chl‐*a*	0.764	Chl‐*a*	0.776	Chl‐*a*	0.798	Chl‐*a*	0.799	Chl‐*a*	0.793	Temp.	0.789
	DO	0.431	Turbidity	0.650	Turbidity	0.636	Turbidity	0.648	Turbidity	0.671	Turbidity	0.672	Turbidity	0.665	CH_4_	0.683
			CH_4_	0.624	CH_4_	0.599	CH_4_	0.620	CH_4_	0.669	CH_4_	0.671	CH_4_	0.655	Turbidity	0.677
			COD	0.524	COD	0.499	COD	0.519	COD	0.568	COD	0.569	COD	0.553	TP	0.466
			T: Lentic	0.425					TP	0.435	TP	0.437	TP	0.404	COD	0.582
									T: Lentic	0.404	T: Lentic	0.406			T: Lentic	0.431
Negative	NH_4_‐N	−0.607	NO_3_‐N	−0.469	NO_3_‐N	−0.454	NO_3_‐N	−0.466	NO_3_‐N	−0.496	NO_3_‐N	−0.496	N NO_3_‐N	−0.487	NO_3_‐N	−0.503

The only group favouring lotic conditions was MOB_I_ (VID = 0.441) in well‐aerated water (VID = 0.431). Notably, MOB_I_ was the only studied group negatively impacted by NH_4_–N (VID = −0.607), while NO_3_–N was the only environmental variable negatively associated with methanogens (VID from −0.454 to −0.503).

## 4 Discussion

Our study highlights the strong variability in microbial community structure and methane fluxes in temperate floodplain lakes, with clear dependence on hydrological connectivity and seasonal thermal dynamics. A pronounced gradient in methane emissions was observed, increasing from lotic to lentic lake types. Lotic oxbows, which maintain regular water exchange with the river, sustained relatively stable dissolved oxygen levels and lower nutrient concentrations, resulting in the lowest CH_4_ and CO_2_ fluxes to the atmosphere (21.7 ± 13.6 mg m^−2^ day^−1^ CH_4_ and 700 ± 500 mg m^−2^ day^−1^ CO_2_). Semi‐lotic oxbows exhibited more variable emissions (225 ± 267 mg m^−2^ day^−1^ CH_4_; 700 ± 400 mg m^−2^ day^−1^ CO_2_), while lentic oxbows—hydrologically isolated from the river—were the most productive in terms of GHG release (507 ± 318 mg m^−2^ day^−1^ CH_4_; 1000 ± 300 mg m^−2^ day^−1^ CO_2_).

These findings are consistent with previous studies showing that methane emissions vary with hydrological isolation, nutrient availability and primary production (Sun et al. 2022; Liu et al. 2022, 2024; Wang, Ge, et al. 2022; Wang, Li, et al. 2022; Khatun et al. 2024; Bastviken et al. 2023). Methane fluxes in floodplain systems are generally higher and more variable than in other lake types, with emissions ranging from 6.9 ± 0.3 mg m^−2^ day^−1^ CH_4_ in subarctic lakes to over 900 mg m^−2^ day^−1^ CH_4_ in highly eutrophic ecosystems like Taihu Lake. In the Olentangy River Wetland Park (Sha et al. 2011), riverside sites emitted between 10.6 and 2742.4 mg m^−2^ day^−1^ CH_4_. Our maximum recorded eCH_4_ value (969 mg m^−2^ day^−1^) occurred in a lentic oxbow during summer, while the lowest (75.6 mg m^−2^ day^−1^) was recorded during winter, reflecting seasonal variability as reported by Hao et al. (2020) and Wang, Ge, et al. (2022), Wang, Li, et al. (2022).

However, temperature alone cannot fully explain the variability in methane fluxes (Bechtold et al. 2025). Other critical drivers include the accumulation of organic matter, sedimentation, periodic anoxia and extensive macrophyte growth—especially in lentic oxbows (Glińska‐Lewczuk 2005, 2009). The high CH_4_ emissions recorded in these systems are likely linked to physical accumulation of methane from deeper, anoxic zones (Kang et al. 2024; Khatun et al. 2024; Klomp et al. 2024), enhanced by sedimentary processes and inputs of organic substrates from macrophytes and allochthonous sources (Bastviken et al. 2023). Macrophytes also facilitate methane production through microhabitat formation, increased organic retention and aerenchymal transport pathways (Covey and Megonigal 2019; Carmichael et al. 2014; Work et al. 2021; Vroom et al. 2022).

The structure of microbial communities involved in methane cycling—methanogens and methanotrophs—varied significantly across both seasons and oxbow types. Methanotrophs (MOB_I_ and MOB_II_) were present throughout the year, but their abundance and functional roles varied by season and habitat. PLS‐R analysis revealed that temperature, CH_4_, chlorophyll‐a, turbidity, COD and TP were key predictors of microbial community composition (Figure 5; Table 4). Methanogenic archaea were positively correlated with lake productivity and elevated CO_2_ levels, particularly in warmer seasons and lentic habitats (West et al. 2016; Yang et al. 2020; Kumar et al. 2021). TOC was a key driver of methanogen abundance, consistent with observations in other freshwater systems (Liu et al. 2024; Khatun et al. 2024; Kang et al. 2024).

Our results also confirmed that methane emissions increased substantially when water temperatures exceeded 20°C, as observed in other studies (Fuchs et al. 2016). In lentic oxbows, CH_4_ concentrations showed strong positive correlations with methanogens (mGen_I–III_: *r* = 0.699–0.807, *p* ≤ 0.0001) and a negative correlation with MOB_I_. While methane production is classically linked to anoxic environments (Wagner 2017; Wang, Ge, et al. 2022; Wang, Li, et al. 2022), recent findings reveal that aerobic methane production occurs in a wide range of organisms and microenvironments, including plants, fungi and cryptogamic covers (Tang et al. 2016; Bižic et al. 2020). In oxygenated surface waters, particularly in the upper layers of reservoirs with low deep‐water methane, this phenomenon is known as the ‘methane paradox’. Contrary to the old belief that methane is produced only without oxygen, it is now known that methane production can happen even in oxygen‐rich waters (Ordóñez et al. 2023). Our study similarly recorded methane supersaturation in oxygenated oxbow lake waters during summer and autumn. This can be attributed to oxygen‐tolerant methanogens and the formation of micro‐anoxic niches within biofilms or microbial mats (Tang et al. 2016; Bižic‐Ionescu et al. 2018).

Methanotrophs serve as critical microbial filters, reducing methane fluxes to the atmosphere. MOB_I_ dominated in well‐oxygenated, dynamic habitats such as lotic and semi‐lotic oxbows. Their ecological plasticity and r‐strategy traits (Steenbergh et al. 2010; Siljanen et al. 2012; Zheng et al. 2024) allow them to function effectively under fluctuating CH_4_ and DO concentrations (Nijman et al. 2021). MOB_II_ were more prevalent in stable, oxygen‐rich and productive conditions—particularly in spring and autumn (Lew and Glińska‐Lewczuk 2018; Wang, Ge, et al. 2022, Wang, Li, et al. 2022; Zheng et al. 2024). Despite lower abundance, their cooperation with MOB_I_ was essential in periods of low CH_4_ flux. In contrast, during summer, high temperatures, competition and predation pressure likely reduced MOB abundance and activity.

To further understand microbial control of methane fluxes, we applied the MOB/mGen ratio as an indicator of the balance between CH_4_ oxidation and production. In lotic systems, high ratios (MOB/mGen > 2) indicated methanotroph dominance and were consistently associated with low CH_4_ fluxes (< 30 mg m^−2^ day^−1^). In contrast, low MOB/mGen ratios observed in lentic oxbow lakes reflected methanogen dominance and corresponded to high CH_4_ emissions, often exceeding 750 mg m^−2^ day^−1^. These patterns were further supported by the results of the GCHAID tree analysis (Figure 4). Based on the observed dataset, we propose a mechanistic framework for predicting CH_4_ flux from microbial community composition and hydrological setting, which effectively differentiates methane‐cycling regimes across systems of varying connectivity (Table 3). Class I environments, with high MOB/mGen ratios and low emissions, reflect strong microbial control. Class II, characterised by balanced microbial state of methanotrophs and methanogens (0.8–2.0), represented a balanced, typically found in semi‐lotic systems and associated with moderate CH_4_ emissions. Classes III and IV are characterised by methanogen‐dominated conditions (MOB/mGen ratios: 0.4–0.8 and < 0.4, respectively) and high emission potential, particularly during summer.

Summer reductions in MOB may also be influenced by zooplankton grazing. Bacterivores such as *Daphnia* spp. are known to significantly suppress methanotroph populations, leading to increased methane fluxes (Taipale et al. 2009; Devlin et al. 2015; Zhao et al. 2023). Even a slight shift in favour of methanogens, under specific conditions (e.g., high temperature, organic enrichment, low DO), resulted in emissions close to 990 mg CH_4_ m^−2^ day^−1^.

The MOB/mGen ratio thus serves as a sensitive and integrative proxy for methane cycling dynamics. Its decline indicates an ecological threshold where methanogenic activity outweighs oxidation capacity, destabilising CH_4_ regulation (Robbins et al. 2016). Despite the conventional view that methane is mainly produced under anoxic conditions, our findings support the inclusion of oxic processes in global methane budgets (Tang et al. 2016; Bižic et al. 2020). The observed imbalance in lentic oxbows highlights their potential as methane hotspots under current and future eutrophication scenarios.

Beaulieu et al. (2019) and Fuchs et al. (2016) emphasise that climate‐driven increases in aquatic productivity may substantially enhance CH_4_ emissions. Modelling studies estimate that eutrophication could raise CH_4_ fluxes by 30%–90%, potentially accounting for 18%–33% of current annual CO_2_ emissions from fossil fuels. To improve the predictive accuracy of methane cycling, future studies should assess microbial metabolic activity at the single‐cell level, with particular focus on archaeal pathways (Dong et al. 2025).

In conclusion, floodplain lakes, especially lentic oxbows, represent important but underrecognized sources of methane. Their contribution to GHG emissions is strongly shaped by microbial dynamics, hydrological connectivity and seasonal shifts. Controlling eutrophication and restoring hydrological exchange in these systems are critical steps for mitigating their impact on atmospheric CH_4_ and maintaining ecosystem stability.

## 5 Conclusion

In summary, our study provides critical insights into the interaction between methanotrophs and methanogens within microbial communities and their roles in regulating methane (CH_4_) and carbon dioxide (CO_2_) emissions from floodplain lakes, based on a case study along the Łyna River in northern Poland. We demonstrated that the degree of hydrological connectivity, along with thermal and trophic conditions, significantly influences these microbial groups and, consequently, CH4 and CO2 emission levels. Particularly, more isolated ecosystems undergo accelerated eutrophication and degradation, contributing substantially to atmospheric CH_4_ and CO_2_ release. Preserving the ecological integrity and homeostasis of floodplain lakes is therefore crucial, as it sustains a diverse and balanced microbiota capable of efficient CH_4_ oxidation, thereby acting as a biological barrier against methane emissions.

Furthermore, our findings emphasise the importance of the MOB to the methanogen (mGen) population ratio as a key indicator of the balance between methane production and consumption. This MOB/mGen ratio offers a reliable metric for assessing the CH_4_ emission potential of aquatic ecosystems and identifying degraded sites where altered biogeochemical and microbiological processes lead to intensified CH_4_ outputs. These results highlight the importance of restoration activities that preserve or re‐establish hydrological connectivity between floodplain lakes and main river channels to mitigate undesirable CH_4_ emissions from these vital wetland systems.

In light of these findings, we advocate for targeted ecological interventions that enhance microbial regulatory functions in floodplain environments, thereby contributing to methane mitigation and broader environmental sustainability goals.

## Author Contributions

**Sylwia Lew:** conceptualization, writing – original draft, investigation, writing – review and editing, methodology, formal analysis, supervision, data curation, visualization. **Paweł Burandt:** data curation, methodology, formal analysis, visualization. **Katarzyna Glińska‐Lewczuk:** conceptualization, investigation, writing – original draft, writing – review and editing, formal analysis, methodology, data curation, visualization.

## Conflicts of Interest

The authors declare no conflicts of interest.

## Data Availability

The datasets used and/or analysed during the current study available from the corresponding author on reasonable request.
